# Linking Plant Community Characteristics and Soil Properties Under Grazing Disturbance in a Desert Steppe Ecosystem

**DOI:** 10.1002/ece3.74444

**Published:** 2026-10-16

**Authors:** Xiao Guan, Rui Dong, Wentao Wang, Yanling Wu

**Affiliations:** ^1^ Department of Ecology, School of Life Science and Technology Inner Mongolia Normal University Hohhot Inner Mongolia Autonomous Region China

**Keywords:** desert steppe, grazing intensity, plant community, soil factors, structural equation modeling

## Abstract

Grazing is a key driver of structural and functional changes in desert steppe ecosystems. Soil nutrients are essential for plant growth, and grazing can alter soil nutrient availability through herbivory and trampling, which, in turn, may affect plant communities. However, the responses of plant communities and their functional groups to soil properties under different grazing intensities remain elusive. This study examined variations in plant community characteristics, including biomass, quantitative traits, and species diversity, under a gradient of grazing intensities. Species were classified into functional groups, and their corresponding soil physicochemical properties were analyzed. The results showed that aboveground biomass was highest under no grazing and light grazing, and lowest under extreme grazing. Height and coverage of annual and biennial forbs and perennial grasses declined sharply with increasing grazing intensity. Species richness and diversity indices reached their minimum values under heavy grazing, while Simpson's dominance index peaked. Soil analysis revealed that available nitrogen and available phosphorus declined with increasing grazing intensity. Pearson correlation analysis revealed positive relationships between available nitrogen, available phosphorus, and plant α‐diversity, biomass, and basic traits. Structural equation modeling further showed that grazing not only directly affected plant communities but also influenced them indirectly through reductions in soil available nitrogen and available phosphorus. In conclusion, regulating grazing intensity to maintain soil nutrient availability may be important for sustaining the stability and functioning of desert steppe ecosystems.

## Graphical abstract

Based on a desert steppe ecosystem, this study constructed four vegetation functional groups, measured their height, coverage, density, and biomass, and determined soil physicochemical properties to explore their interrelationships.

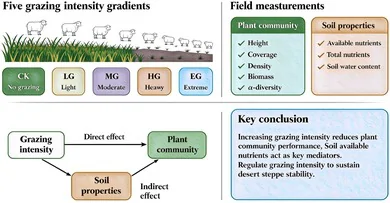

## 1 Introduction

Grasslands, vital ecosystems of the terrestrial biosphere (Estell et al. 2012), cover approximately 25% of the Earth's land surface and provide substantial ecological (Adams et al. 1990), economic, and societal benefits. In China, grasslands cover approximately 2.6 × 10^8^ ha (National Bureau of Statistics 2021), accounting for 41.7% of the national territory (Ministry of Ecology and Environment 2008) and constitute a key element of the country's terrestrial ecosystems (Xu et al. 2019). Among these, desert steppe constitutes the most arid grassland type along the grassland‐desert transition zone (Hou et al. 2024). Characterized by sparse vegetation and poor soil conditions, desert steppes are highly vulnerable to disturbance and are particularly susceptible to degradation under grazing pressure (Sun et al. 2018; Wang et al. 2022). This vulnerability is especially evident in Sonid Right Banner, central Inner Mongolia (Chen et al. 2023), where sheep grazing is the dominant land use and grazing‐induced disturbance significantly affects vegetation dynamics.

Grazing, as a principal mode of grassland utilization, influences ecosystems through herbivory and trampling, directly affecting vegetation and indirectly altering soil physicochemical properties by changing ground surface conditions (Sekar et al. 2024; Shao et al. 2013). Livestock excretions (urine and feces) are also important nutrient inputs into grazed grassland soils (Bardgett and Wardle 2003). Previous studies have demonstrated that increasing grazing intensity generally reduces plant community and functional group basic traits (e.g., height, coverage, and density), α‐diversity (Wang et al. 2016), and plant biomass (Hou et al. 2024). However, the relationship between grazing intensity and plant diversity is not simply linear. A global meta‐analysis revealed that the response of species richness and diversity to grazing intensity depends on aridity: in dry areas, even light grazing can reduce plant diversity, whereas in humid areas, moderate grazing may maintain or enhance diversity (Zhang et al. 2023). Another global synthesis found that the effects of grazing on plant biomass and biodiversity are strongly moderated by climate and grazing patterns (Cao et al. 2024).

Plant functional groups, defined as assemblages of species sharing similar adaptive traits, life‐history strategies, and ecological roles, provide an effective framework for understanding vegetation responses to environmental change (Wright and Francia 2024). This framework is useful because species within the same functional group tend to respond similarly to grazing owing to shared morphological traits (e.g., plant height, palatability, and belowground storage organs) and regeneration strategies (e.g., annual vs. perennial life cycles) (Cingolani et al. 2005). Grazing acts as a selective filter on these traits: tolerant groups may persist or expand, whereas sensitive groups are suppressed.

In desert steppe ecosystems, which are specifically characterized by low productivity and high environmental stress, plant communities are particularly sensitive to grazing pressure, and diversity often declines continuously with increasing intensity rather than showing a hump‐shaped response. Evidence suggests that heavy grazing significantly decreases the aboveground biomass of all functional groups, weakens the dominance of perennial grasses, increases the relative abundance of perennial forbs, and may even induce shrub encroachment in semi‐arid steppes, thereby fundamentally altering ecosystem structure and function (Lei et al. 2024; Zhu et al. 2025). Additionally, high grazing intensity tends to increase the spatial aggregation of dominant species, resulting in more pronounced vegetation patchiness (Hou et al. 2024; Li et al. 2024).

Soil, as the primary substrate for plant growth, plays a critical role in mediating vegetation responses. Grazing‐induced changes in soil properties can, therefore, feedback to influence plant communities. Studies have shown that grazing intensity creates clear gradients in soil nutrients within desert steppes: with increasing grazing pressure, available nitrogen and phosphorus in the surface soil decline, while available potassium exhibits a unimodal response; total phosphorus and potassium remain relatively stable but decrease significantly under heavy grazing (Meng et al. 2024; Wright 2005). Soil moisture is particularly sensitive to grazing, with light grazing enhancing soil porosity and water retention, whereas heavy grazing markedly reduces water content (Wang et al. 2021). These findings suggest that moderate grazing may help maintain soil nutrient balance and water availability, whereas overgrazing diminishes both soil fertility and water‐holding capacity (Lei et al. 2023).

Despite extensive research on plant functional groups and soil properties, comparative analyses of the responses of individual species versus functional groups to grazing intensity remain limited. Furthermore, the interactions between functional groups and environmental factors under grazing disturbance remain poorly understood. Two recent studies based on the same experimental platform examined grazing effects on plant diversity (Xue et al. 2025) and soil nematode communities (Dong et al. 2025). However, neither study systematically quantified the integrated relationships among functional groups, plant community traits, and soil physicochemical properties across a complete grazing gradient (from no grazing to extreme grazing).

In arid and semi‐arid desert steppe regions, prolonged overgrazing has disrupted ecosystem stability and poses a serious threat to sustainable pastoralism (Li et al. 2008). However, the relationships among plant communities, their functional groups, and soil factors in these systems remain insufficiently understood. Therefore, using the desert steppe of Sonid Right Banner in Xilingol League, Inner Mongolia, as a case study, this research aimed to quantify variations in vegetation and soil physicochemical properties under different grazing regimes. Specifically, the objectives were to (i) compare the responses of whole plant communities and individual functional groups to grazing intensity and (ii) elucidate the influence of soil factors on community structure and functional‐group dynamics across grazing gradients.

## 2 Materials and Methods

### 2.1 Site Description

The study was conducted in a desert steppe at Hardenhushu Gacha, Inner Mongolia, China (113°10′26.92″ E, 42°21′09.2″ N), at an elevation of approximately 1350 m above sea level (a.s.l.) (Figure 1). The region experiences a temperate semi‐arid continental climate, characterized by a large annual temperature range, with recorded extremes from 38.7°C to −38.8°C, and a frost‐free period of approximately 130 days.

**FIGURE 1:**
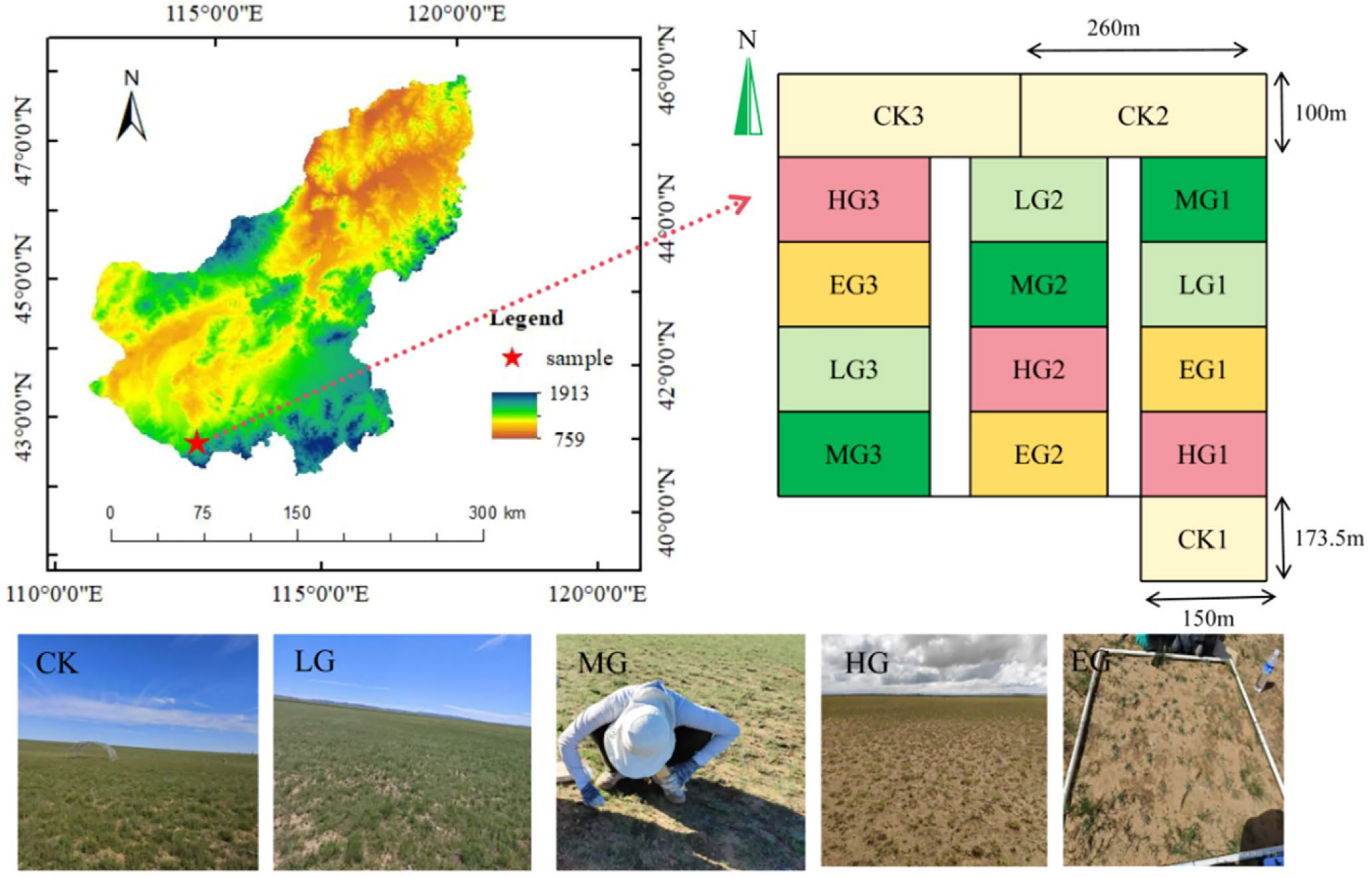
Spatial distribution of experimental plots within the desert steppe study area.

From 2020 to 2023, the mean annual temperature was 5.97°C, with a mean annual precipitation of 180 mm and evaporation of 2384 mm. The soil is classified as gray‐brown calcic soil according to the Chinese Soil System Classification (CSSC), with an approximate thickness of 30 cm, a sandy texture, and a pH of 7.12.

The study area lies within the *Stipa breviflora* steppe, where *Stipa breviflora* Griseb is the dominant species, playing a dominant role in shaping community structure and ecosystem functioning. Other dominant species include *Cleistogenes songorica* (Roshev.) Ohwi and *Allium polyrhizum* Turcz., both of which exhibit strong grazing tolerance and high palatability.

### 2.2 Experimental Design

The experiment followed a completely randomized block design with five treatments and three replicates, yielding a total of 15 plots (each 2.6 ha). The treatments were as follows: no grazing (CK, 0 sheep·ha^−1^ per year; control with 0 sheep); light grazing (LG, 1.54 sheep·ha^−1^ per year; i.e., 4 sheep per plot); moderate grazing (MG, 1.92 sheep·ha^−1^ per year; i.e., 5 sheep per plot); heavy grazing (HG, 2.31 sheep·ha^−1^ per year; i.e., 6 sheep per plot); and extreme grazing (EG, 2.69 sheep·ha^−1^ per year; i.e., 7 sheep per plot) (Figure 1) (Dong et al. 2025). These stocking rates span the range from light to heavy grazing relative to the regional grazing intensity gradients (0.91–2.71 sheep units·ha^−1^·half year^−1^, including light, moderate, and heavy grazing) established for this ecosystem (Lv et al. 2026).

The plots were separated by fenced boundaries to minimize potential interference between adjacent plots, and each plot was grazed independently at its designated stocking rate. The grazing treatments were established in 2010 and maintained annually from June to October. Data for this study were collected in August 2024. The 14‐year duration of the treatments allowed sufficient time for treatment‐specific effects to develop, and the clear gradient responses observed across treatments provide empirical evidence that the treatment effects were distinguishable despite the spatial proximity of the plots. Similar approaches using adjacent plots as independent experimental units are common in rangeland grazing studies (Lai and Kumar 2020).

### 2.3 Vegetation and Soil Sampling

Five 1 × 1 m quadrats were randomly established in each plot, yielding a total of 75 quadrats in August 2024. Within each quadrat, plant species composition, height, coverage, and density were recorded. Aboveground biomass was harvested, air‐dried, and weighed. Plant height was measured using a ruler, with 5–10 randomly selected individuals measured per species in each quadrat. Coverage was visually estimated as the percentage of ground area covered by each species. Density was determined by counting the individuals of each species within the quadrat. All plant species recorded in the quadrats were classified into four functional groups based on life form and growth habit: (1) annual and biennial forbs, (2) perennial grasses, (3) shrubs and semi‐shrubs, and (4) perennial forbs. This classification followed previous studies of desert steppe vegetation in Inner Mongolia (Ma et al. 2013).

Before soil sampling, aboveground plant material and surface litter were carefully removed from each sampling point to avoid contamination. Soil samples were collected from the topsoil layer (0–10 cm) using an auger at three sampling points per quadrat within each of the five quadrats per plot. The 0–10 cm layer was selected because it is the primary zone of root activity and nutrient cycling in this desert steppe ecosystem, and grazing‐induced changes in soil properties are most pronounced in this surface layer.

### 2.4 Soil Physicochemical Analysis

Soil water content was determined using the oven‐drying method. One fresh soil subsample was oven‐dried at 105°C for 24 h to constant weight to determine soil water content. The second subsample was air‐dried, passed through a 1 mm sieve for analysis of available potassium, available phosphorus, and available (alkali‐hydrolysable) nitrogen, and further through a 0.149 mm mesh for total phosphorus and total potassium analyses.

Available potassium was extracted with NH_4_OAc and measured by flame photometry. Available phosphorus was extracted using 0.5 M NaHCO_3_ (Olsen method) and determined spectrophotometrically using a U‐T3C UV–Vis spectrophotometer (Summit Instrument Manufacturing (Shanghai) Co. Ltd., China). Available nitrogen was measured using the alkaline diffusion method, which measures ammonium‐N only. Total phosphorus and total potassium were determined after NaOH fusion digestion, using the molybdenum‐antimony colorimetric method and flame photometry, respectively. All analyses followed standard procedures described in *Soil Agro‐Chemical Analysis* (Bao 2000).

### 2.5 Data Processing

Plant community diversity was evaluated using the following indices:

(1) Species richness (Sobs): the number of species recorded in each quadrat.

(2) Simpson Index

$$D=1-\sum_{i=1}^{S}{P_{i}}^{2}$$

(3) Shannon–Wiener index

$$H=-\sum_{i=1}^{S}P_{i}\ln P_{i}$$

(4) Pielou index:

$$J=\frac{H}{\ln S}$$

where *p_i_* denotes the proportion of the density of plant species, *i* is the total density in the plant community, and *n* is the number of plant species.

The Importance Value (IV) of each species was calculated as the average of three relative measures: relative height, relative coverage, and relative density. These components were calculated as follows:

1. Relative height = (sum of heights of a given species in the quadrat/sum of heights of all species) × 100%
2. Relative coverage = (coverage of a given species/total coverage of all species) × 100%
3. Relative density = (density of a given species/total density of all species) × 100%
4. IV = (relative height + relative coverage + relative density)/3

For functional‐group‐level analyses, the IV of each functional group was calculated by averaging the IV values of all species belonging to that group. Species IV was calculated within each quadrat and then averaged across the five quadrats within each plot; data were not pooled across quadrats before calculating relative values.

### 2.6 Statistical Analysis

All statistical analyses were conducted in R version 4.3.2.

A multi‐level analytical framework was used to evaluate the relationships among grazing intensity, plant communities, and soil properties, in which ANOVA, correlation analysis, Mantel tests, and SEM were applied sequentially to address different levels of ecological questions.

Plant community α‐diversity indices were calculated using Mothur (version 1.30.2). Differences among treatments were tested using one‐way ANOVA followed by Duncan's multiple range test (*p <* 0.05). The bar chart was generated using GraphPad Prism 10.

Pearson correlation analysis was performed to examine relationships between soil physicochemical properties and (i) plant α‐diversity, (ii) aboveground biomass, and (iii) key plant functional traits, and results were visualized using the *corrplot* package.

Mantel tests (via the *vegan* package) were used to evaluate the strength and significance of relationships among the four variable groups: plant α‐diversity, plant biomass, plant basic traits, and soil physicochemical properties. A partial least squares structural equation modeling (PLS‐SEM) approach, implemented with the *plspm* package, was used to assess how grazing intensity influences plant communities through soil properties. Plant response variables included plant α‐diversity (PD), aboveground biomass (PB), and basic traits (BT). PD was constructed as a latent variable using four indicators: species richness (Sobs), Shannon–Wiener index, Simpson's reciprocal index, and Pielou's evenness index. Sobs reflects species number, Shannon reflects both richness and evenness, Simpson emphasizes dominance, and Pielou measures evenness, together providing a more comprehensive description of community α‐diversity than any single indicator alone. BT was also constructed as a latent variable using four community‐level indicators: height, coverage, density, and importance value. Soil variables included available nutrients (AN, comprising available nitrogen, available phosphorus, and available potassium), total nutrients (TN, comprising total phosphorus and total potassium), and soil water content (SWC). Model performance was evaluated using the goodness‐of‐fit (GoF) index.

## 3 Results

### 3.1 Effects of Grazing on the Plant Communities

#### 3.1.1 Effect of Grazing on Basic Traits

As shown in Table 1, increasing grazing intensity induced pronounced and divergent responses across the four plant functional groups in height, coverage, density, and importance value (IV).

**TABLE 1:** Basic traits (height, coverage, density, and importance value) of different plant functional groups under varying grazing intensities.

BOC	GI	ABF	SS	PG	PF
Height (cm)	CK	4.90 ± 0.63a	3.25 ± 0.91a	7.33 ± 0.88a	2.32 ± 0.20a
	LG	1.17 ± 0.26b	2.04 ± 0.59ab	6.42 ± 1.14a	1.94 ± 0.29a
	MG	0.38 ± 0.09b	0.89 ± 0.33b	3.38 ± 0.76b	1.27 ± 0.19b
	HG	0.67 ± 0.19b	0.42 ± 0.2b	3.46 ± 0.74b	0.73 ± 0.13b
	EG	0.23 ± 0.07b	0.96 ± 0.34b	1.71 ± 0.26b	0.76 ± 0.16b
Coverage (%)	CK	3.34 ± 0.98a	0.90 ± 0.25a	7.94 ± 1.45a	1.48 ± 0.25a
	LG	0.26 ± 0.11b	0.97 ± 0.35a	5.13 ± 0.61b	0.70 ± 0.14b
	MG	0.12 ± 0.04b	0.21 ± 0.08b	3.54 ± 0.29bc	0.68 ± 0.08b
	HG	0.31 ± 0.17b	0.07 ± 0.04b	3.43 ± 0.35bc	0.63 ± 0.11b
	EG	0.03 ± 0.01b	0.15 ± 0.06b	2.32 ± 0.24c	0.55 ± 0.14b
Density (plants m^−2^)	CK	2.38 ± 0.77a	0.90 ± 0.21ab	3.70 ± 0.65a	6.83 ± 0.83b
	LG	0.96 ± 0.31b	1.42 ± 0.42a	5.33 ± 1.01a	7.75 ± 0.95ab
	MG	0.54 ± 0.19b	0.52 ± 0.18b	5.45 ± 0.82a	11.92 ± 2.85a
	HG	0.94 ± 0.38b	0.25 ± 0.15b	5.93 ± 1.05a	9.17 ± 0.73ab
	EG	0.27 ± 0.08b	0.52 ± 0.21b	4.02 ± 0.85a	5.96 ± 0.51b
Importance value	CK	26.96 ± 5.40a	11.12 ± 2.48ab	33.06 ± 3.93a	28.86 ± 3.00b
	LG	7.44 ± 1.68b	14.73 ± 3.69a	36.76 ± 4.53a	36.41 ± 4.80ab
	MG	4.68 ± 1.22b	8.31 ± 3.18ab	46.84 ± 4.79a	40.22 ± 2.83a
	HG	11.32 ± 3.85b	4.14 ± 1.96b	41.96 ± 4.17a	42.58 ± 2.25a
	EG	4.72 ± 1.88b	12.13 ± 3.95ab	45.11 ± 6.00a	38.04 ± 4.5ab

*Note:* Values are presented as mean ± standard error. Different lowercase letters within the same column indicate significant differences among grazing intensities (*p* < 0.05).

Abbreviations: ABF, annual and biennial forbs; BOC, basic traits; CK, no grazing; EG, extreme grazing; GI, grazing intensity; HG, heavy grazing; LG, light grazing; MG, moderate grazing; PF, perennial forbs; PG, perennial grasses; SS, shrubs and semi‐shrubs.

Plant height and coverage of annual and biennial forbs and perennial grasses were highest in the control (no grazing) and lowest under extreme grazing. Specifically, height decreased by 92% and 71%, while coverage declined by 99% and 71%, respectively. Shrubs and semi‐shrubs, as well as perennial forbs, exhibited significantly greater height and coverage in the no grazing than in the other treatments (*p* < 0.05).

For plant density, annual and biennial forbs reached their maximum in no grazing (*p* < 0.05), whereas shrubs and semi‐shrubs peaked under light grazing (*p* < 0.05). Perennial grasses showed no significant variation across treatments, while perennial forbs were significantly more abundant in the moderate grazing treatment than in the extreme grazing (*p* < 0.05).

Annual and biennial forbs exhibited the highest IV in no grazing (*p* < 0.05), whereas shrubs and semi‐shrubs showed the lowest under heavy grazing (*p* < 0.05). Perennial grasses showed no significant differences in IV among treatments, whereas perennial forbs exhibited slightly higher values in moderate grazing and heavy grazing than in no grazing (*p* < 0.05).

#### 3.1.2 Effects of Grazing on Plant Biomass

The effects of grazing on plant biomass are illustrated in Figure 2. Total aboveground biomass declined significantly with increasing grazing intensity. Biomass in no grazing and light grazing treatments was significantly higher than in moderate, heavy, and extreme grazing, exceeding that in extreme grazing by 86% and 71%, respectively. Biomass of annual and biennial forbs and perennial forbs was significantly higher in no grazing than in other treatments (*p* < 0.05). Biomass of perennial grasses in no grazing and light grazing was significantly higher than in moderate, heavy, and extreme grazing (*p* < 0.05). In contrast, shrubs and semi‐shrubs reached their highest biomass under light grazing, with significant differences from moderate, heavy, and extreme grazing (*p* < 0.05).

**FIGURE 2:**
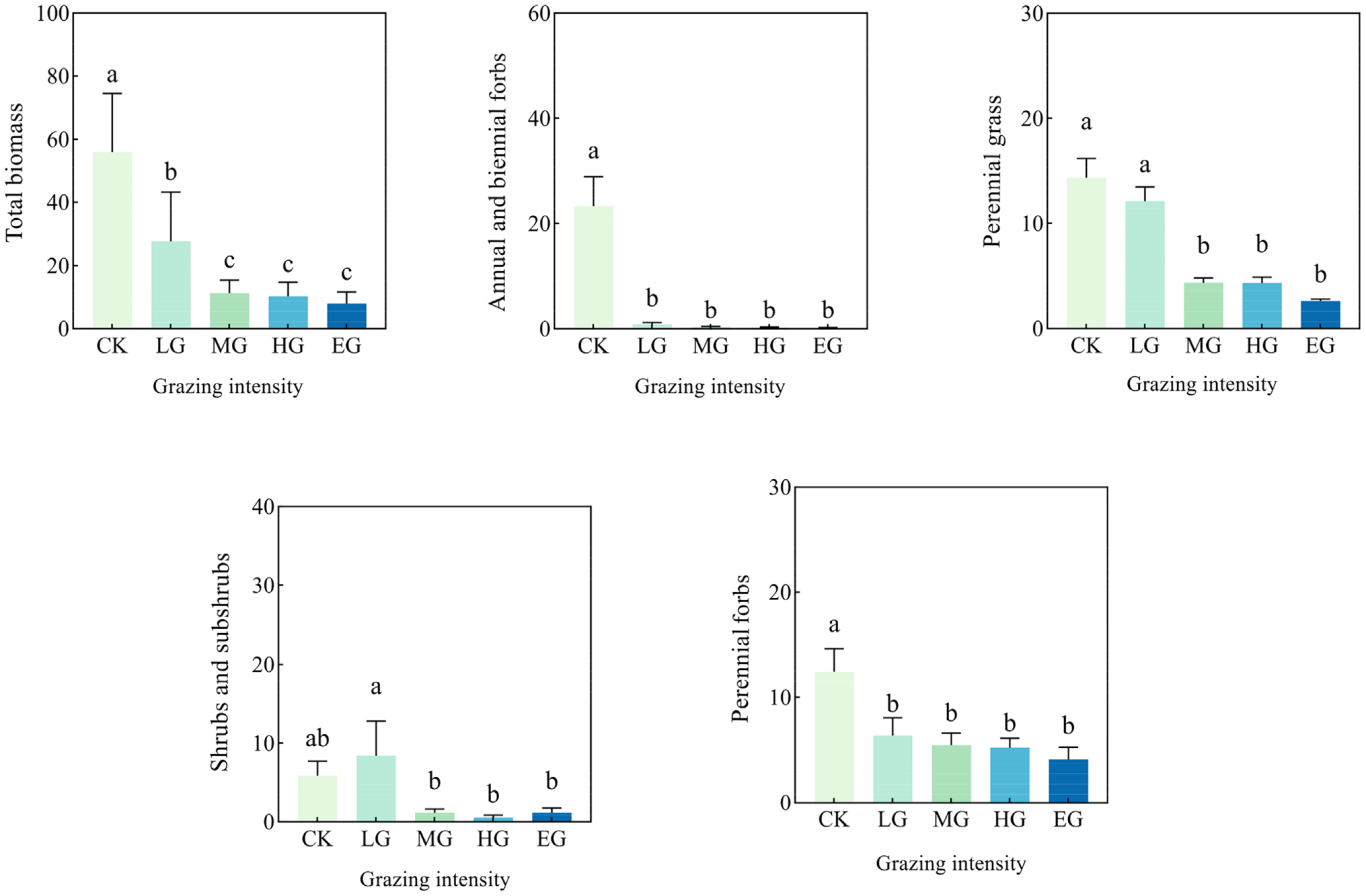
Effects of grazing intensity on total biomass and the biomass of different plant functional groups (annual and biennial forbs, perennial grasses, shrubs and semi‐shrubs, and perennial forbs) in the desert steppe. CK, (no grazing); LG, light grazing; MG, moderate grazing; HG, heavy grazing; EG, extreme grazing. Values are presented as the mean ± standard error. Different lowercase letters indicate significant differences among grazing intensities (*p* < 0.05).

#### 3.1.3 Effects of Grazing on Plant α‐Diversity

The effects of grazing on plant α‐diversity are shown in Figure 3. Species richness (Sobs), Shannon‐Wiener, and Pielou indices generally decreased with increasing grazing intensity, reaching their lowest values under heavy grazing. The Sobs index was highest in light grazing, whereas Shannon‐Wiener and Pielou indices were highest in no grazing, with all three indices decreasing under heavy grazing by 14%, 29%, and 36%, respectively (*p* < 0.05). In contrast, Simpson's reciprocal index was highest under heavy grazing treatment and significantly higher than in no grazing and light grazing (*p* < 0.05). These results indicate that heavy grazing reduces species richness and evenness while increasing community dominance.

**FIGURE 3:**
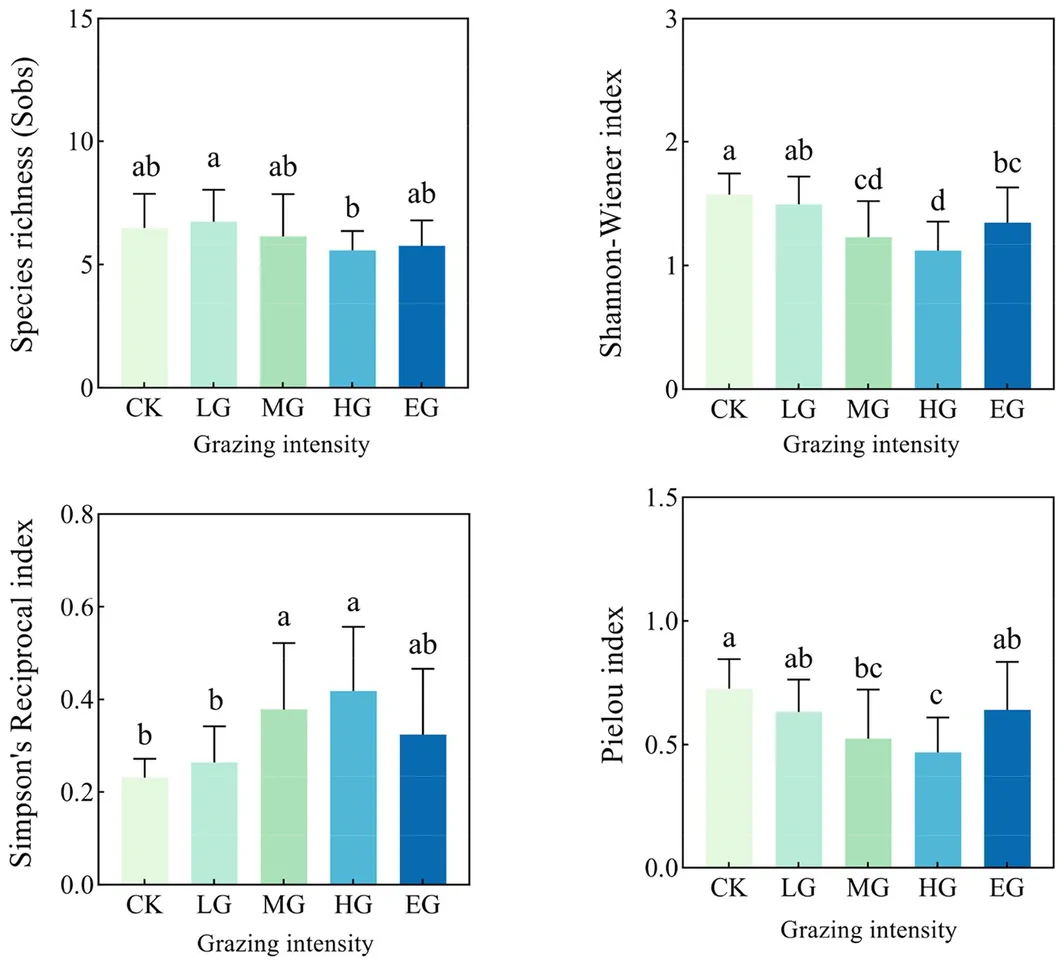
Effects of grazing intensity on plant α‐diversity indices, including species richness (Sobs), Shannon‐Wiener index, Simpson's reciprocal index, and Pielou's evenness index, in the desert steppe. CK, (no grazing); LG, light grazing; MG, moderate grazing; HG, heavy grazing; EG, extreme grazing. Values are presented as the mean ± standard error. Different lowercase letters indicate significant differences among grazing intensities (*p* < 0.05).

#### 3.1.4 Effects of Grazing on Soil Physicochemical Properties

The effects of grazing on soil physicochemical properties are shown in Figure 4. Most soil available nutrients decreased with increasing grazing intensity, whereas total nutrients showed no significant variation across grazing intensities. Notably, the declines in available potassium and available nitrogen were particularly pronounced. The available potassium content in the no grazing treatment was 230.15 mg kg^−1^, significantly higher than in the other treatments (*p* < 0.05), with reductions of 12%, 11%, 10%, and 15%, respectively. Compared with no grazing, the available nutrient content was significantly reduced under heavy and extreme grazing (*p* < 0.05) by approximately 26%.

**FIGURE 4:**
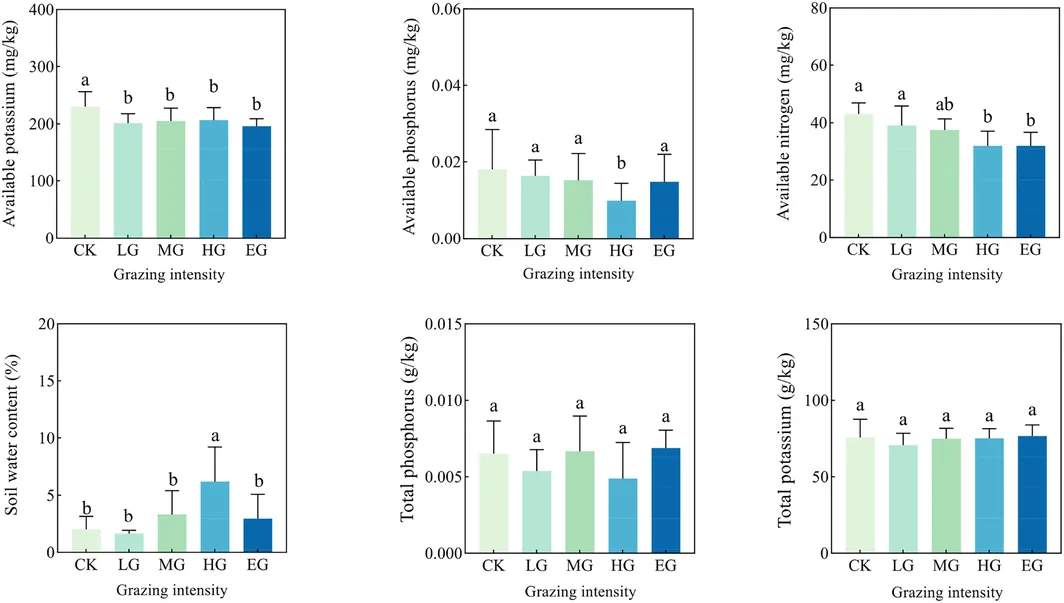
Effects of grazing intensity on soil physicochemical properties. Bar charts show the influence of five grazing intensities (CK: No grazing; LG: Light grazing; MG: Moderate grazing; HG: Heavy grazing; EG: Extreme grazing) on soil properties. Panels represent: (a) available potassium (mg kg^−1^), (b) available phosphorus (mg kg^−1^), (c) available nitrogen (mg kg^−1^), (d) soil water content (%), (e) total phosphorus (g kg^−1^), and (f) total potassium (g kg^−1^). Bars indicate mean values ± standard error. Different lowercase letters above bars denote significant differences among grazing intensities (*p* < 0.05).

Available phosphorus exhibited a unimodal response, initially decreasing and then increasing with grazing intensity, reaching its minimum under heavy grazing. Compared with no grazing, available phosphorus in heavy grazing decreased by 45% (*p* < 0.05). In contrast, soil water content exhibited the opposite trend, rising initially and then declining, with a maximum of 6% under heavy grazing, significantly higher than in the other treatments (*p* < 0.05).

### 3.2 Relationships Between Plant Communities and Soil Properties

Pearson correlation analysis (Figure 5a) revealed strong associations between soil nutrients and plant community characteristics.

**FIGURE 5:**
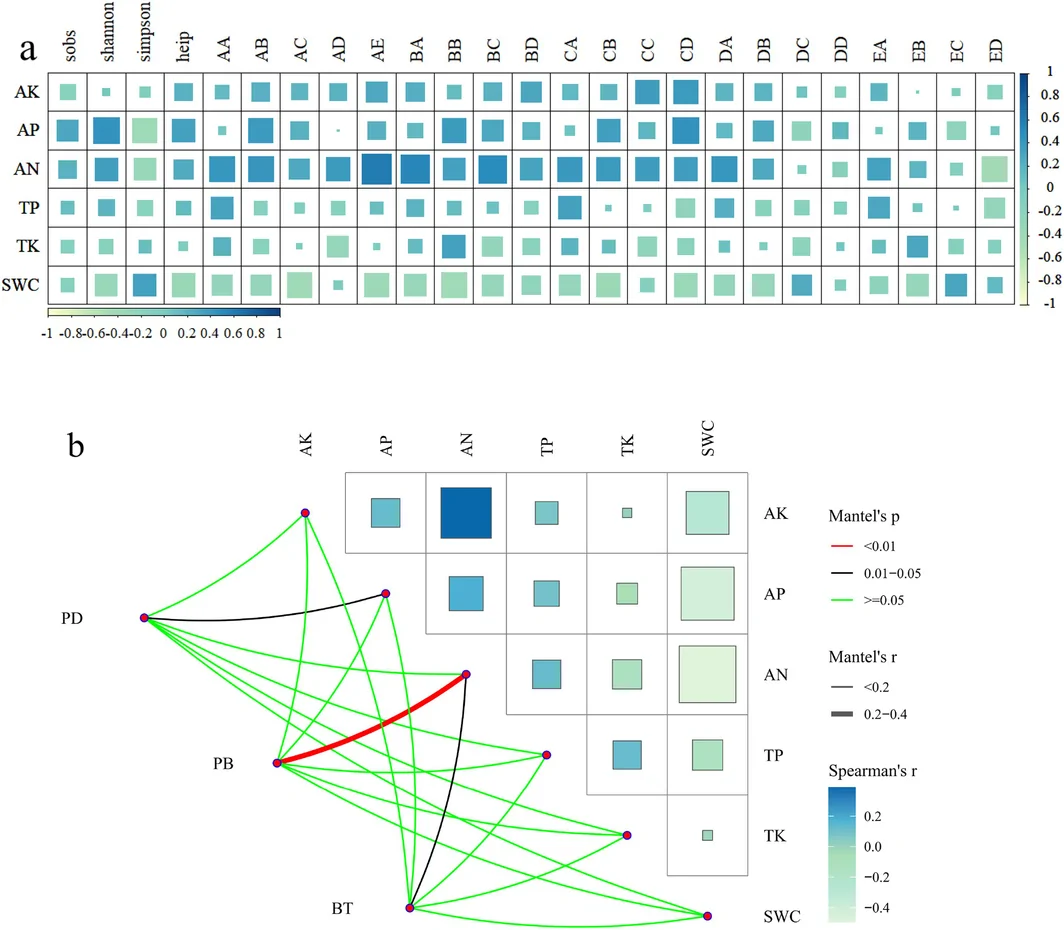
(a, b) Relationships between plant communities and soil physicochemical properties under different grazing intensities (a). (a) The heatmap shows Spearman's correlation coefficients between plant community composition, including plant α‐diversity, plant biomass (AA‐AE), height (BA‐BD), coverage (CA‐CD), density (DA‐DD), importance value (EA‐ED), and soil physicochemical properties, including available potassium (AK), available phosphorus (AP), available nitrogen (AN), total phosphorus (TP), total potassium (TK), and soil water content (SWC). Color gradients represent the strength and direction of correlations (blue: Positive; green: Negative), and square size indicates the magnitude of the correlation coefficient. (b) Network diagram illustrating the relationships between plant diversity indices (PD: Plant α‐diversity; PB: Plant biomass; BT: Basic traits) and soil physicochemical properties. Line colors indicate significance levels based on Mantel tests (red: *p* < 0.01; gray: 0.01–0.05; green: *p* > 0.05), while line thickness represents the strength of Mantel's *r*. Square sizes and colors within the matrix correspond to Spearman's correlation coefficients between soil variables.

Available nitrogen was significantly positively correlated with the Shannon‐Wiener index and negatively correlated with Simpson's dominance index (*p* < 0.05). It also showed significant positive relationships with the biomass of multiple functional groups (*p* < 0.05) and with total biomass (*p* < 0.01). Additionally, available nitrogen was significantly positively correlated with several basic traits, including height, coverage, density, and importance value across functional groups.

Available phosphorus exhibited a highly significant positive correlation with the Shannon‐Wiener index (*p* < 0.01) and significant positive correlations with Simpson dominance and Pielou evenness indices (*p* < 0.05). It was also significantly positively associated with shrub and semi‐shrub biomass and structural traits, as well as annual and biennial forb density (*p* < 0.05).

In contrast, available potassium and soil water content showed relatively weaker relationships with plant community characteristics. Available potassium was significantly and positively correlated only with the coverage of perennial grasses and forbs (*p* < 0.05). In contrast, soil water content showed significant positive associations with Simpson's index, Pielou's evenness index, perennial grass biomass, total biomass, and shrub and semi‐shrub height (*p* < 0.05).

Neither total phosphorus nor total potassium showed any significant correlations with plant community variables. Overall, available nitrogen and available phosphorus emerged as the primary drivers of plant α‐diversity, biomass, and community traits, with available nitrogen having the strongest influence.

Mantel test results were consistent with these findings (Figure 5b), showing that plant α‐diversity was significantly and positively associated with available phosphorus (*p* < 0.05), and plant biomass was highly significantly and positively correlated with available nitrogen (*p* < 0.01). Basic community traits were significantly and positively related to available nitrogen (*p* < 0.05).

### 3.3 Integrated Analysis of Plant Community‐Soil Relationships

Structural equation modeling (SEM) (Figure 6) was used to evaluate the direct and indirect effects of grazing on basic traits in plant communities. The model explained 43.96% of the variation in the response variables.

**FIGURE 6:**
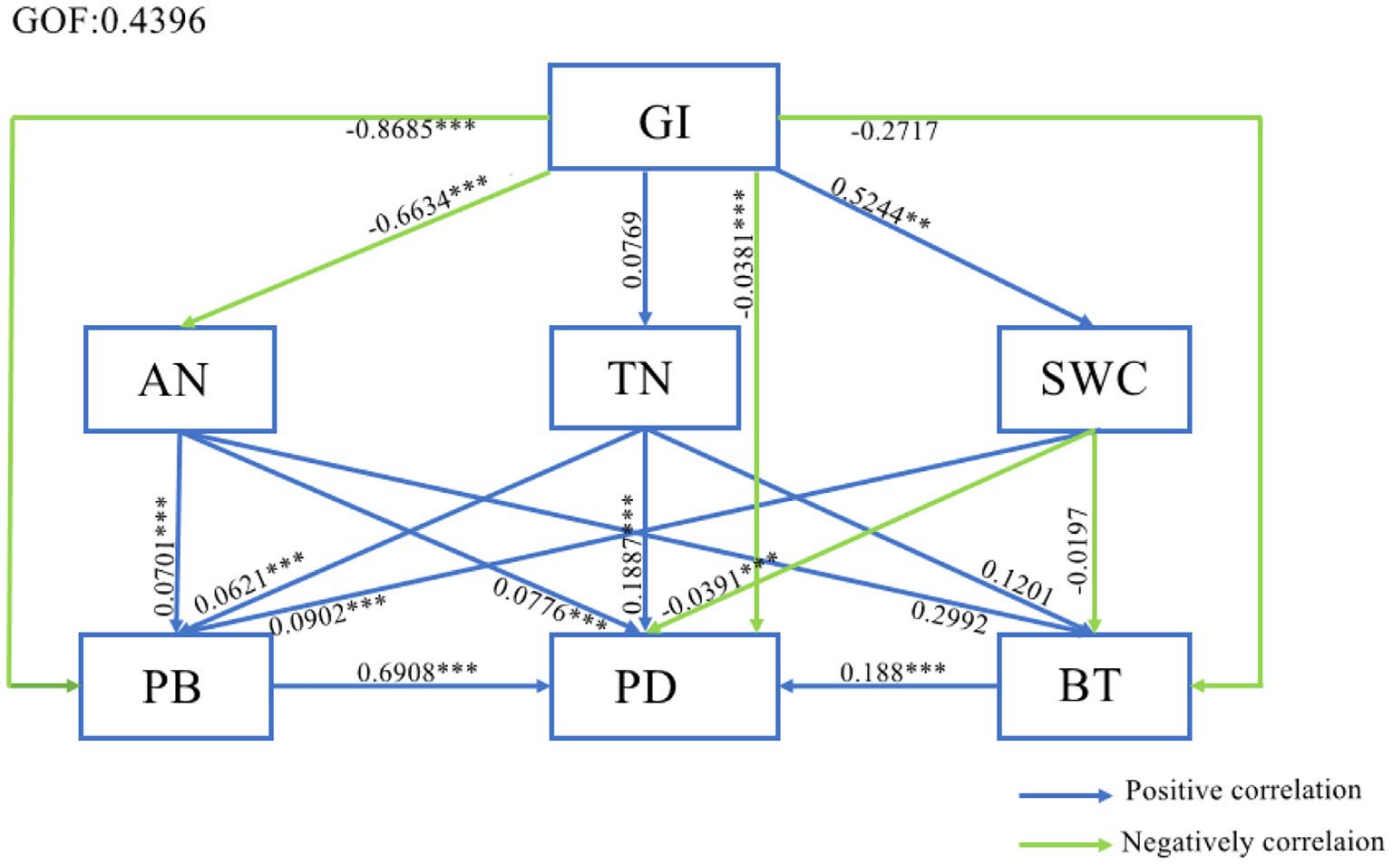
Structural equation model (SEM) depicting the associations between grazing intensity, soil properties, and plant community attributes. The model depicts the associations between grazing intensity and plant communities and soil properties. PD and BT are latent variables. PD was indicated by species richness (Sobs), Shannon‐Wiener index, Simpson's reciprocal index, and Pielou's evenness index, and BT was indicated by height, coverage, density, and importance value. Blue and green arrows indicate positive and negative relationships, respectively. Numbers adjacent to arrows are standardized path coefficients. Asterisks denote statistical significance (*p < 0.05, **p < 0.01, ***p < 0.001). GOF = 0.4396. AN, Available nutrients (available nitrogen, available phosphorus, and available potassium); GI, Grazing intensity; PB, Plant biomass; SWC, Soil water content; TN, Total nutrients (total phosphorus and total potassium).

Grazing intensity had no significant direct effect on plant α‐diversity, but it significantly reduced available nitrogen and available phosphorus (*p* < 0.05) while increasing soil water content (*p* < 0.05). Given the significant positive effect of available phosphorus on α‐diversity (*p* < 0.05), grazing indirectly reduced diversity by negatively influencing available phosphorus.

For plant biomass and basic community traits, grazing exerted both direct and indirect effects via changes in soil properties. However, the direct effects were stronger, with highly significant negative impacts on both biomass and community traits (*p* < 0.01). This indicates that grazing intensity is the dominant driver of variation in plant biomass and basic community characteristics.

## 4 Discussion

### 4.1 Responses of Basic Traits of Plant Communities to Grazing Disturbance

The progressive decline in aboveground biomass with increasing grazing intensity observed in this study is partially consistent with the grazing optimization hypothesis, which proposes that moderate grazing can stimulate compensatory growth and maintain productivity, whereas heavy grazing suppresses biomass accumulation through repeated defoliation and reduced photosynthetic capacity (Ferraro and Oesterheld 2002; Zhu et al. 2025).

The four functional groups exhibited divergent response patterns. Annual and biennial forbs and perennial grasses‐the most palatable groups‐showed the greatest sensitivity, with sharp declines in height, coverage, and importance value even under light grazing. Shrubs and semi‐shrubs displayed an inverted U‐shaped response: their density peaked under light grazing and declined thereafter, suggesting a temporary release from competition with taller herbs under moderate disturbance (Zainelabdeen et al. 2021). Under heavy grazing, however, the importance value of shrubs dropped to its minimum, indicating that once grazing pressure exceeds the regenerative capacity of shrubs, the system may approach a critical threshold. Perennial forbs increased in importance value under moderate and heavy grazing, largely driven by the expansion of degradation‐indicator species such as *Convolvulus ammannii* (Zhao et al. 2023). This asymmetric response among functional groups‐particularly the contrasting trajectories of palatable grasses versus unpalatable forbs and shrubs‐has not been systematically documented in previous studies from this region, which have typically focused on community‐level responses without comparing functional group dynamics across a complete grazing gradient.

### 4.2 Responses of Plant Community α‐Diversity to Grazing Disturbance

Plant α‐diversity exhibited a unimodal response to grazing intensity, reaching its minimum under heavy grazing and showing a slight recovery under extreme grazing. This pattern is consistent with the competitive exclusion principle, whereby dominant species monopolize limited resources under heavy pressure, excluding subordinate species and reducing overall diversity (Huisman 1994).

The slight recovery of diversity under extreme grazing may reflect two mechanisms. First, extreme grazing intensity can increase habitat heterogeneity through vegetation fragmentation, creating microhabitats that allow stress‐tolerant and trampling‐resistant species to persist (Wu et al. 2011). Second, under extreme disturbance, the competitive dominance of palatable species is reduced so severely that subordinate species, which are often less palatable or more tolerant of grazing, gain access to resources from which they would otherwise be excluded. This nonmonotonic diversity response—particularly the distinction between heavy and extreme grazing—has not been systematically documented in previous studies from this region, which typically treated heavy and extreme grazing as a single intensity level (Xue et al. 2025).

### 4.3 Responses of Soil Physicochemical Properties to Grazing Disturbance

Soil physicochemical properties are critical indicators of soil quality (Zhang et al. 2021). Among the soil properties examined, available nitrogen and available phosphorus showed the most pronounced responses to grazing intensity. Available nitrogen declined progressively with increasing grazing pressure, reflecting reduced plant biomass and root exudation under heavy grazing, which weakens nutrient retention and increases nitrogen losses (Fan et al. 2023; Yang et al. 2016). Available phosphorus exhibited a U‐shaped response, reaching its minimum under heavy grazing and recovering slightly under extreme grazing. This pattern likely reflects the combined effects of reduced root exudate inputs (which weaken phosphorus adsorption) and potential microbial‐mediated activation of residual phosphorus under extreme disturbance (Bicharanloo et al. 2022; Devi et al. 2024). Available potassium also declined with increasing grazing intensity, though its response was less pronounced than that of available nitrogen and available phosphorus.

Soil water content showed the opposite trend, peaking under heavy grazing. While grazing‐induced compaction generally reduces infiltration and water‐holding capacity, the extremely low vegetation cover under heavy grazing may have reduced transpiration, offsetting the reduction in infiltration and resulting in elevated surface soil water content (Dong et al. 2012).

The identification of available nitrogen and available phosphorus as the primary soil variables associated with plant community changes represents a key contribution of this study. Previous studies in this region have often treated soil properties as a homogeneous set of responses to grazing (Jiao et al. 2016), without systematically distinguishing between available and total nutrients or identifying the specific soil variables most closely associated with changes in plant diversity, biomass, and functional‐group composition.

Historically, the *Stipa breviflora* desert steppe was part of the Mongolian Plateau grassland ecosystem, where wild ungulates such as the Mongolian gazelle ( *Procapra gutturosa* ) and argali sheep ( *Ovis ammon* ) were widely distributed during the Pleistocene and Holocene. Traditional nomadic pastoralism has also been practiced in this region for millennia, with livestock moving seasonally between pastures (Liu et al. 2019). The grazing‐intensity gradient in this experiment (0–2.69 sheep·ha^−1^·year^−1^) was established based on the stocking‐rate standards recommended by local pastoral management authorities and previous studies conducted on the same long‐term grazing platform (Wang et al. 2023). Heavy grazing corresponds to actual conditions in overgrazed rangelands, whereas extreme grazing was included to assess ecosystem responses beyond currently recognized degradation thresholds. Even if heavy grazing may exceed natural grazing intensities during some historical periods, these treatments provide valuable insights: they reveal degradation thresholds and critical transitions in community structure under sustained high‐intensity grazing and help assess the potential for recovery of heavily degraded grasslands following grazing reduction, which is relevant to current “livestock–grassland balance” policies and ecological restoration practices (Li et al. 2017).

### 4.4 Mechanisms of Soil Factor Effects on Desert Steppe Plant Communities Under Grazing Disturbance

Plant–soil feedback is a key driver of desert steppe community dynamics. Soil physicochemical properties regulate the distribution of plant functional groups and strongly influence community structure and diversity by mediating interspecific competition and regeneration processes (Kulmatiski et al. 2008). In this study, Pearson correlation, Mantel tests, and structural equation modeling consistently showed that available nitrogen and available phosphorus were the primary soil properties associated with changes in plant community attributes. Both available nitrogen and available phosphorus were positively associated with plant α‐diversity, biomass, and basic traits, which is consistent with previous studies of desert steppe ecosystems (Liu and Shao 2024; Zhao et al. 2025). Greater nitrogen availability promotes root expansion and biomass accumulation, whereas greater phosphorus availability improves phosphorus uptake efficiency and facilitates seed germination. Together, these effects explain their positive associations with community productivity and diversity (Güsewell 2004; Vitousek et al. 2010).

The SEM analysis further indicated that grazing intensity was more strongly associated with plant biomass and basic traits through direct pathways than through soil‐mediated indirect pathways. This result suggests that the primary mechanism by which grazing alters plant communities in this desert steppe is the direct removal of aboveground biomass and modification of plant traits through herbivory and trampling, with soil nutrient depletion playing a secondary but still significant mediating role.

These findings extend previous work in this region. Whereas earlier studies based on the same experimental platform separately examined the effects of grazing on plant diversity and soil nematode communities (Dong et al. 2025), our results indicate that direct grazing pressure and declining soil nutrients‐particularly reductions in available nitrogen and available phosphorus—jointly contributed to the observed changes in community structure and function.

A limitation of this study is that the findings are based on sampling conducted during a single year, and site‐specific meteorological data were unavailable for the 2024 sampling year. Although all treatments were exposed to the same climatic conditions, the possibility that interannual climatic variation influenced the observed patterns cannot be fully excluded. Long‐term monitoring is needed to determine whether these patterns persist over time and to better understand the feedback mechanisms between plant functional groups and soil properties that control desert steppe community succession.

## 5 Conclusions

With increasing grazing intensity, plant biomass and the basic traits of all four functional groups declined, with biomass showing the most sensitive response to grazing disturbance. Plant community α‐diversity exhibited a unimodal pattern, decreasing initially and then increasing with grazing intensity. Pearson correlation and Mantel tests identified soil available nitrogen (AN) and available phosphorus (AP) as the soil properties most strongly associated with plant community characteristics, with their decline closely linked to reduced community productivity. Structural equation modeling further suggested that grazing is associated with both direct and indirect associations with plant communities through reductions in soil nutrient availability (AN and AP). Overall, regulating grazing intensity to maintain soil nutrient supply may be important for sustaining the stability and functioning of desert steppe ecosystems.

## Author Contributions

**Xiao Guan:** conceptualization (lead), data curation (lead), formal analysis (lead), investigation (lead), writing – original draft (lead). **Yanling Wu:** funding acquisition (equal), investigation (equal), supervision (equal), writing – review and editing (equal). **Rui Dong:** conceptualization (lead), data curation (equal), formal analysis (lead), methodology (lead), supervision (equal), writing – review and editing (equal). **Wentao Wang:** visualization (equal).

## Funding

This work was supported by the National Natural Science Foundation of China (31860156), and Inner Mongolia Natural Science Foundation (2026MS0862).

## Conflicts of Interest

The authors declare no conflicts of interest.

## Supporting information


**Data S1:** ece374444‐sup‐0001‐supinfo.xlsx.

## Data Availability

The data that support the findings of this study are available in the Supporting Information of this article.
